# Snow Surface Microbiome on the High Antarctic Plateau (DOME C)

**DOI:** 10.1371/journal.pone.0104505

**Published:** 2014-08-07

**Authors:** Luigi Michaud, Angelina Lo Giudice, Mohamed Mysara, Pieter Monsieurs, Carmela Raffa, Natalie Leys, Stefano Amalfitano, Rob Van Houdt

**Affiliations:** 1 Department of Biological and Environmental Sciences, University of Messina, Messina, Italy; 2 Unit of Microbiology, Belgian Nuclear Research Centre (SCK•CEN), Mol, Belgium; 3 Department of Bioscience Engineering, Vrije Universiteit Brussel, Brussels, Belgium; 4 Water Research Institute, National Research Council (IRSA-CNR), Rome, Italy; National Cancer Institute, NIH, United States of America

## Abstract

The cryosphere is an integral part of the global climate system and one of the major habitable ecosystems of Earth's biosphere. These permanently frozen environments harbor diverse, viable and metabolically active microbial populations that represent almost all the major phylogenetic groups. In this study, we investigated the microbial diversity in the surface snow surrounding the Concordia Research Station on the High Antarctic Plateau through a polyphasic approach, including direct prokaryotic quantification by flow cytometry and catalyzed reporter deposition fluorescence *in situ* hybridization (CARD-FISH), and phylogenetic identification by 16S RNA gene clone library sequencing and 454 16S amplicon pyrosequencing. Although the microbial abundance was low (<10^3^ cells/ml of snowmelt), concordant results were obtained with the different techniques. The microbial community was mainly composed of members of the *Alpha-proteobacteria* class (e.g. *Kiloniellaceae* and *Rhodobacteraceae*), which is one of the most well-represented bacterial groups in marine habitats, *Bacteroidetes* (e.g. *Cryomorphaceae* and *Flavobacteriaceae*) and *Cyanobacteria*. Based on our results, polar microorganisms could not only be considered as deposited airborne particles, but as an active component of the snowpack ecology of the High Antarctic Plateau.

## Introduction

The cryosphere, the portion of Earth's biosphere where water is in solid form as snow or ice, includes vast areas of sea ice, freshwater ice, glaciers, ice sheets, snow cover and permafrost [1]. The extremely harsh climatic conditions act as a severe ecological filter for all immigrant and resident organisms and represent the best analogue on Earth for the search of extraterrestrial life [2], [3]. Cold/frozen environments harbour abundant and diverse microorganisms, which are not merely repositories for wind-transported microorganisms [4]. In particular snow ecological systems, which cover about 35% of the Earth's surface permanently or for varying times during the year [1], constitute dynamic reservoirs of nutrients and microorganisms [5].

Studies on the cryosphere revealed the presence of a number of bacteria (mainly belonging to the *Bacteroidetes*, *Actinobacteria*, *Firmicutes*, and *Proteobacteria*) [6], which must cope with a combination of severe environmental stressors including (ultra)oligotrophic conditions, high solar UV radiation, freeze/thaw cycles, and limited liquid water [4]. Carpenter *et al.* [7] retrieved from the South Pole snow sequences related to *Thermus-Deinococcus*-like organisms, and reported low rates of bacterial DNA and protein synthesis. Junge *et al.* [8] reported that bacterial activity can occur at subzero temperatures. Recently, Lopatina *et al.* [9] retrieved from the snow surrounding Antarctic coastal Russian Stations some bacterial genera (*Variovorax*, *Janthinobacterium*, *Pseudomonas*, and *Sphingomonas*) that may be considered as endogenous Antarctic snow inhabitants.

Several physiological characteristics allow prokaryotes to survive in these harsh environments such as spore formation, pigmentation, increased membrane fluidity and production of enzymes active at low temperature [10].

Interest in the survival mechanisms of microorganisms in cold environments is increasing because of the possibility to discover new sources of biotechnologically interesting molecules and of the desire to understand the boundaries of life on Earth or elsewhere in our solar system. The evidence of ice on Mars and Europa has made the isolation and study of psychrophiles particularly important for determining the types of organisms that can survive in frozen environments [11].

The Dome C, situated on top of the Antarctic Plateau (3 233 m above sea level), is one of the coldest and driest places on Earth with temperatures that hardly rise above −25°C in summer (annual average air temperature is −54.5°C). Only a few reports on the diversity of Antarctic Plateau-associated microorganisms exist, although the diversity of Antarctic prokaryotes has been extensively studied [12], [13].

The aim of the present work was to explore the prokaryotic diversity and source of microorganisms in the surface snow (corresponding to the annual deposition) of a pristine site on the Dome C, near the Italian-French Research base Concordia. A polyphasic approach, including direct prokaryotic identification by epifluorescence, catalyzed reporter deposition fluorescence *in situ* hybridization (CARD-FISH), flow-cytometry, 16S rRNA gene clone library sequencing and high-throughput 454 16S amplicon pyrosequencing was adopted.

## Materials and Methods

### Sampling and preliminary treatment of samples

**Figure 1:**
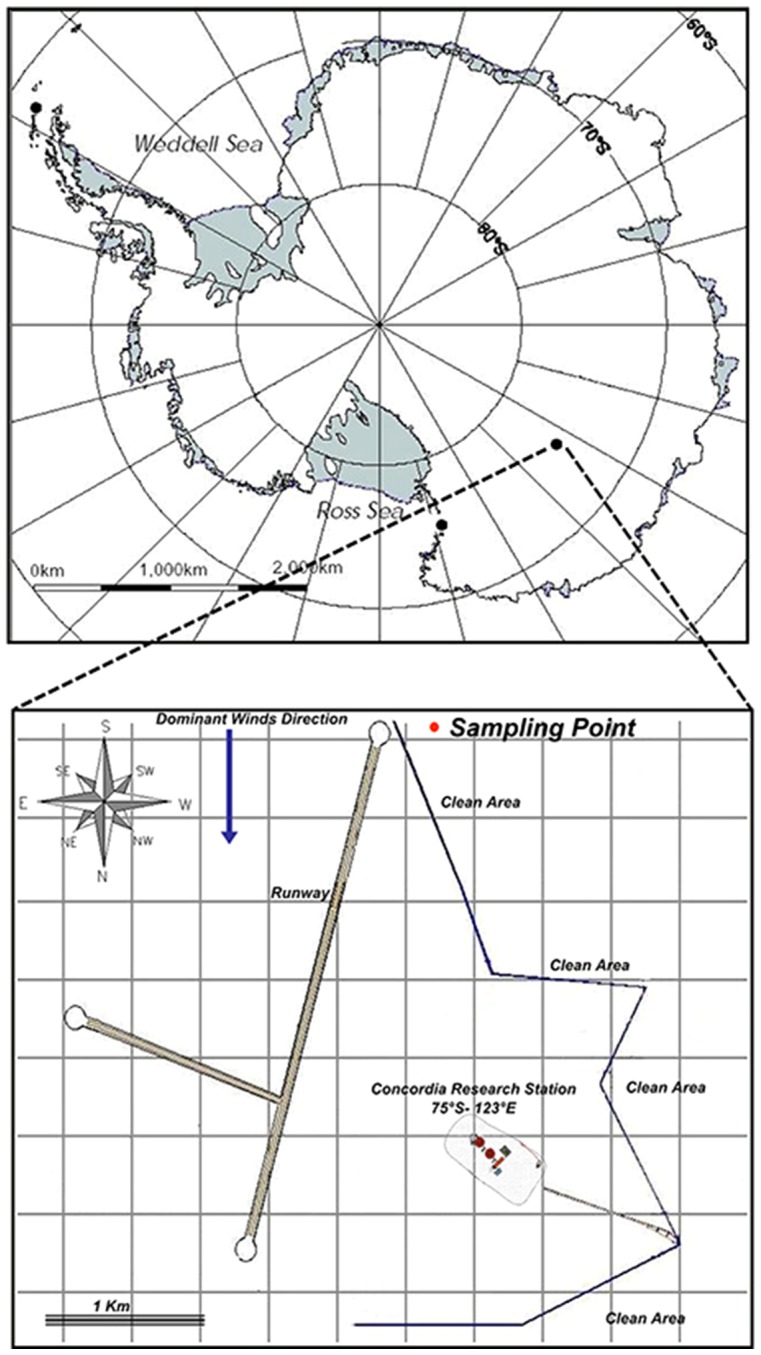
Concordia Research Station location and sampling point.

Snow surface sample (top 10 cm corresponding to the annual deposition) was collected in triplicate from a “Clean Area” 2 km from the Research Base “Concordia” (75°06′S–123°20′E) (Fig. 1). No specific permissions were required for sampling activities. Field studies did not involve endangered or protected species.

Snow temperature was −31°C, while the air temperature was −29°C with a wind chill of −45.6°C. The snow was very compact with the presence of extended wavelike sharp and irregular ridges of hard snow (termed “sastrugi”) [4].

Sampling was performed by using polyethylene boxes pre-treated with 1M hydrogen chloride and hydrogen peroxide, as previously described [14]. Sterile gloves and suit, and an ethanol flame-sterilized shovel were used. Collected samples were allowed to thaw at 4°C for 24–48 h in the laboratory facility of the Base (one hundred litres of packed snow *per* sample resulted in approximately 20 litres of snowmelt). Autoclave-sterilized Milli-Q water was treated in tandem with the snow samples as a negative-control field blank.

Melted snow samples were processed as follows: a) for total counting and CARD-FISH, 500 ml was fixed with formalin, incubated at 4°C for 1 h and stored at −20°C; b) for flow cytometry analysis, 5 ml was fixed with a freshly prepared solution of 1% paraformaldehyde and 0.05% glutaraldehyde (final concentrations) and then stored at −20°C; c) for DNA extraction, 15 l was filtered through a 0.2-µm-pore-size Sterivex filter unit (Millipore). The filters were stored at −20°C in lysis buffer (50 mM tris, 40 mM EDTA, and 750 mM sucrose).

Samples were packed for shipment and maintained refrigerated at −20°C during the trip back to Europe. Sample temperature was constantly monitored and registered by c\temp™ single-use data logger (Marathon Products Inc.).

### Total microbial cell counts by fluorescence microscopy and flow cytometry

For microscopic observation, samples were filtered through 0.2-µm black 25-mm-diameter polycarbonate filters (Isopore) and stained with 4′,6-diamidino-2-phenylindole (DAPI) (1 µg ml^−1^, final concentration; Sigma) for 10 min. Filters were mounted on slides with non-fluorescent mounting oil (Immersol 518 M). Subsequently, cell enumeration by epifluorescence microscopy was carried out at magnification 1000× by using an Axioplan (Zeiss) fluorescence microscope [15].

Microbial cells in the snowmelt samples were quantified and characterized by the Flow Cytometer A50-micro (Apogee Flow System, Hertfordshire, England) equipped with a solid state laser set at 20 mV and tuned to an excitation wave length of 488 nm. The volumetric absolute counting was carried out by staining with SYBR Green I (1∶10000 final concentration; Molecular Probes, Invitrogen), while the red autofluorescent cell signals, due to photosynthetic pigments, were used to identify and quantify photoautotrophic microorganisms. Thresholding was carried out using the green or red channel, respectively. The light scattering signals (forward and side light scatter), green fluorescence (535/35 nm), orange fluorescence (590/35 nm) and red fluorescence (>610 nm) were acquired for cell group discrimination. Samples were run at a flow rate of 38.9 µl min^−1^, in order to keep the number of total events below 1000 event/s. An exclusion gate was applied to avoid visualization of abiotic particles characterized by low green and high red fluorescence. Since cell numbers were expected to be around the detection limit of the instrument, repeated runs were computed together to reach at least 300 positive cells in the counting gate. Data were analyzed using the Apogee Histogram Software (v2.05).

### CARD-FISH

The relative abundance of bacterial groups was examined by performing CARD-FISH reactions with bacteria-specific probes (EUB338 mix), an Archaea-specific probe (ARCH915) and 14 different bacterial group-specific probes (Table 1) [16]–[26]. The probe NON338 was used as a negative control [27]. Samples (100 ml) were filtered at low pressure (200 mbar) onto 0.22 µm white polycarbonate filters (type GTTP; diameter, 47 mm, Millipore). Filters were cut in sections and dipped in 0.2% (w/v) low-gelling point agarose (gel strength 300 g cm^−2^, Biozym, USA), and then treated as described in Pernthaler *et al.* [28], [29] and Teira *et al.* [30]. Filter sections were counterstained with DAPI at a final concentration of 1 µg ml^−1^ and mounted on a glass slide with Citifluor and Vectashield in a 4∶1 proportion. Slides were stored at −20°C. CARD-FISH filter sections were visualized under a Zeiss epifluorescence microscope. Cell count was done by enumerating at least 500 DAPI-stained cells in 20 fields covering an area of 100 µm×100 µm each and numbers were compared to probe-stained cells in the same field.

**Table 1:** HRP labelled oligonucleotide probes used in this study.

Probe^a^	Target group	Probe sequence (5′-3′)	Reference
EUB338I	Most but not all Bacteria	GCTGCCTCCCGTAGGAGT	[16]
EUB338II	*Planctomycetes*	GCAGCCACCCGTAGGTGT	[17]
EUB338III	*Verrucomicrobiales*	GCTGCCACCCGTAGGTGT	[17]
ALF968	*Alphaproteobacteria*	GGTAAGGTTCTGCGCGTT	[18]
BET42a	*Betaproteobacteria*	GCCTTCCCACTTCGTTT	[19]
GAM42a	*Gammaproteobacteria*	GCCTTCCCACATCGTTT	[19]
DELTA495a	Most *Deltaproteobacteria*	AGTTAGCCGGTGCTTCCT	[20]
DELTA495b	*Deltaproteobacteria*	AGTTAGCCGGCGCTTCCT	[20]
DELTA495c	*Deltaproteobacteria*	AATTAGCCGGTGCTTCCT	[20]
EPSY914	*Epsilonproteobacteria*	GGTCCCCGTCTATTCCTT	[21]
CF319a	*Bacteroidetes*	TGGTCCGTGTCTCAGTAC	[22]
HGC69a	*Actinobacteria* (high G+C content−Gram+bacteria)	TATAGTTTACCACCGCCGT	[23]
LGC354a	*Firmicutes* (low G+C content−Gram+bacteria)	TGGAAGATTCCCTACTGC	[24]
LGC354b	*Firmicutes* (low G+C content−Gram+bacteria)	CGGAAGATTCCCTACTGC	[24]
LGC354c	*Firmicutes* (low G+C content−Gram+bacteria)	CCGAAGATTCCCTACTGC	[24]
CYA664	Most *Cyanobacteria*	GGAATTCCCTCTGCCCC	[25]
CYA762	Most *Cyanobacteria*	CGCTCCCCTAGCTTTCGTC	[25]
ARCH915	*Archaea*	GTGCTCCCCCGCCAATTCCT	[26]
NON338	Negative control probe	ACTCCTACGGGAGGCAGC	[27]

a Probes EUB338I, EUB338II and EUB338III were equimolarly mixed together to obtain the EUB-mix; the probes LGC354a, LGC354b and LGC354c were equimolarly mixed together to obtain the LGC-mix; Probes DELTA495a, DELTA495b and DELTA495c were equimolarly mixed together to obtain the DELTA-mix; Probes CYA664 and CYA762 were equimolarly mixed together to obtain the CYA-mix.

### DNA extraction

Total genomic DNA from melted snow was extracted in triplicate using the phenol-chloroform method according to Zhou *et al.* [31], precipitated by adding 0.7 volumes of 100% isopropanol followed by a wash with ice-cold 70% ethanol, and after air-drying resuspended in 50 µl of deionizated sterile water. Quantity and quality of extracted DNA was checked by nanodrop ND-1000 device and the Quant-iT PicoGreen dsDNA reagent and kit (Life Tech, Carlsbad, USA) following the manufacturer's instructions.

### 454 16S amplicon pyrosequencing

PCR of a bacterial 16S rRNA gene fragment (V1–V3 region, 507 bp) and subsequent tag-encoded pyrosequencing were performed at DNAVision (Charleroi, Belgium). The 16S rRNA genes were amplified using the two universal primers 8F (5′- AGAGTTTGATCCTGGCTCAG -3′) and 518R (5′- ATTACCGCGGCTGCTGG -3′). The forward primer contained the sequence of the Titanium A adaptor (5′-CCATCTCATCCCTGCGTGTCTCCGACTCAG-3′) and a barcode sequence. For each sample, a PCR mix of 100 µl was prepared containing 1×PCR buffer, 2U of KAPA HiFi Hotstart polymerase blend and dNTPs (Kapabiosystems), 300 nM primers (Eurogentec, Liege, Belgium), and 60 ng gDNA. Thermal cycling consisted of initial denaturation at 95°C for 5 min, followed by 25 cycles of denaturation at 98°C for 20 s, annealing at 56°C for 40 s, and extension at 72°C for 20 s, with a final extension of 5 min at 72°C. 3 µl of PCR product were added to a new PCR mix (identical as first round of PCR) for the nested PCR of 15 cycles. Amplicons were visualized on 1% agarose gels using GelGreen Nucleic Acid gel stain in 1× TAE (Biotium) and were cleaned using the Wizard SV Gel and PCR Clean-up System (Promega) according to the manufacturer's instructions. Pyrosequencing was carried out using the forward primer on a 454 Life Sciences Genome Sequencer FLX instrument (Roche) following titanium chemistry.

These pyrosequencing data have been submitted to the Sequence Read Archive of NCBI under the accession number SRX518097, BioProjectID number PRJNA244715.

### 16S rRNA gene clone library

Extracted DNA from triplicate samples was pooled and firstly used for clone libraries for Bacteria and Archaea. All PCR amplifications were performed with an ABI9600 thermocycler (PE; Applied Biosystems).

For amplification of the bacterial 16S rRNA gene the forward primer 8f (5′-AGAGTTTGATCCTGGCTCAG-3′) and the reverse primer 907r (5′-CCGTCAATTCCTTTRAGTTT-3′) were used. The PCR program and reaction conditions are described in Michaud *et al.* [13].

For archaeal 16S rRNA gene amplification two different protocols were tested: a) primers 340f (5′-CCCTAYGGGGYGCASCAG-3′) and 1000r (5′-GAGARGWRGTGCATGGCC-3′) [32] with GoTaq Green Master Mix (Promega); b) primers Arc20f (5′-GTTTCCGGTTGATCCYGCCRG-3′) and Arc958r (5′-GTTTYCCGGCGTTGAMTCCAATT-3′) [33] with Takara's Ex *Taq* DNA Polymerase. No amplification of archaeal 16S rRNA genes was detected.

The bacterial 16S rRNA gene library was constructed using the products of five PCR reactions that were pooled and ligated into the pGEM Easy Vector System (Promega) according to the manufacturer's instructions. The resulting ligation products were used to transform *Escherichia coli* ElectroMAX DH10B cells (Invitrogen). Inserts were subsequently PCR amplified from lysed white colonies using vector-specific primers, M13f (5′-GTAAAACGACGGCCAGT-3′) and M13r (5′-CAGGAAACAGCTATGACC-3′), under the same previously described PCR conditions [13]. Results were analyzed by agarose gel electrophoresis as described previously [13]. The amplified 16S rRNA gene fragments were directly purified from PCR reaction mixture using the QIAquick PCR Purification Kit (Qiagen) according to the supplier's instructions. Automated sequencing of the 16S rRNA gene clone library was carried out by Sanger sequencing using the dye-terminator method (Macrogen, Korea). Sequence data have been submitted to the GenBank database under accession Nos KJ870937-KJ870986.

### Analysis of 16S rRNA gene clone library and 454 amplicon pyrosequencing data

Reads produced by Sanger sequencing and 454 pyrosequencing were trimmed using the mothur software [34] as described below. 454 generated sequences containing more than two errors in the primer sequence, or more than one error in the barcode sequence were removed. Pyrosequencing reads and Sanger reads were first subjected to basic trimming as recommended [35] via the removal of reads with read length lower than 200 nts, containing one or more ambiguous bases, or having a homopolymer longer than 8 (only applied for pyrosequencing data). Next, a quality trimming approach was applied using a sliding window of 100 nt length with a cut-off on the Phred score of 30 and 20 for pyrosequencing and Sanger reads, respectively. For Sanger, we used the TraceTuner software (3.0.6; http://sourceforge.net/projects/tracetuner/) in order to convert the quality files in Phred score. All reads were aligned to the Silva reference database (http://www.mothur.org/wiki/Silva_reference_alignment), thereby removing reads starting extremely late, or ending extremely early in the alignment (belong in the 2% outliers of the start and end) using the mothur filter and screen commands. Pyrosequencing reads were denoised using NoDe (Mysara *et al.*, unpublished data; Noise Detector (NoDe) is an artificial intelligence-based classifier that first identifies positions likely to be erroneous and subsequently clusters those error-prone reads with correct reads resulting in error-free consensus reads.), while the Sanger sequencing data were denoised using the SLP algorithm [36] as implemented in mothur, using a maximum of 2% differences. Chimera removal and operational taxonomic unit (OTU) clustering of the pyrosequencing reads was performed using UPARSE [37]. For the Sanger sequencing data we used the UCHIME chimera detection tool [38] together with nearest neighbour clustering algorithm implemented in mothur (with 0.03 distance). Phylotyping was performed using the RDP classifier [39] retrained with the greengenes dataset (using 80% confidence threshold as cut off), as recommended in [35].

### Phylogenetic tree construction

For each phylotype the most representative read was selected by calculating the pairwise distance between all reads in a single Phylotype and selecting the read with the smallest maximum distance to all other reads (mothur Get.Oturep command), which led to 23 and 178 reads for the clone library and pyrosequencing data, respectively. For the pyrosequencing reads, only the 15 most abundant phylotypes were selected in order to improve readability of the tree. The closest relatives of these phylotypes were obtained using the RDP classifier [39] with the RDP cultured and the greengenes dataset, leading to 33 and 34 unique hits, respectively. The full set of sequences was aligned against the SILVA database and the resulting alignments were cleaned (using the align.ses and filter.seqs commands as available in mothur [34]). The distance was calculated and parsed for tree creation using mothur (dist.seqs and clearcut commands) [34]. Visualization of the phylogenetic tree was done using FigTree [http://tree.bio.ed.ac.uk/software/figtree/].

### Scanning electron microscopy

For scanning electron microscopy (SEM) samples were gently thawed at 4°C and filtered on a 0.02 µm (25 mm diameters) inorganic membrane (*Anodisc*; Whatman). Filters were fixed with glutaraldehyde (4%) in 0.1% cacodylate buffer at pH 7.4 for 3 h and then dehydrated with 25, 50, 70, 85, and 95% ethanol for 5 min. Subsequent process manipulation and photography were carried out using a SEM JEOL JSM-6500F (JEOL-USA Inc., Peabody, MA USA).

## Results

### Prokaryotic and pico-eukaryotic abundances

Direct counts by epifluorescence microscopy revealed population densities of 4.3±2.2×10^2^ cells ml^−1^ of snowmelt (mean ± standard deviation). In turn, 1.7±0.6×10^2^ autofluorescent cells ml^−1^ of snowmelt were detected.

**Figure 2:**
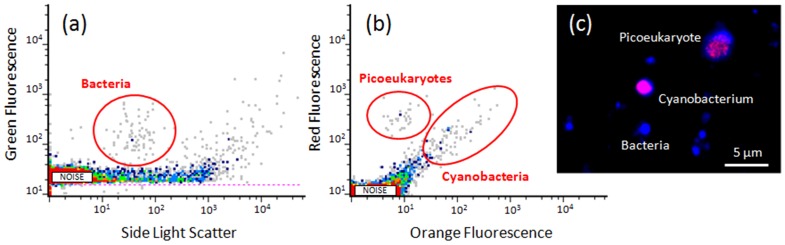
Representative cytograms. Total prokaryotes (a), Cyanobacteria and picoeukaryotes (b) retrieved in the snowmelt samples by SYBR Green I staining and autofluorescence signals, respectively. The microscopic control confirmed their presence according to cell fluorescence and size (c).

Flow cytometry showed a comparable number of non-autofluorescent prokaryotes in the snowmelt samples (2.5±0.5×10^2^ cells ml^−1^) (Fig. 2a). An abundance of 0.9±0.3×10^2^ cyanobacterial cells ml^−1^ (26.5% of the total counted prokaryotes) was determined based on the autofluorescent signals in an orange-*vs*-red cytograms (Fig. 2b). The autofluorescent signals were coherent with those of cells containing phycocyanin and phycoerythrin. A further cytometric population of photoautotrophs showed red fluorescence and forward scatter signals similar to those of picoeukaryotes, but the exact number was not properly determinable by flow cytometry. However, a qualitative microscopic control confirmed the presence of a few picoeukaryotic cells in the snowmelt sample (Fig. 2c).

### CARD-FISH

**Figure 3:**
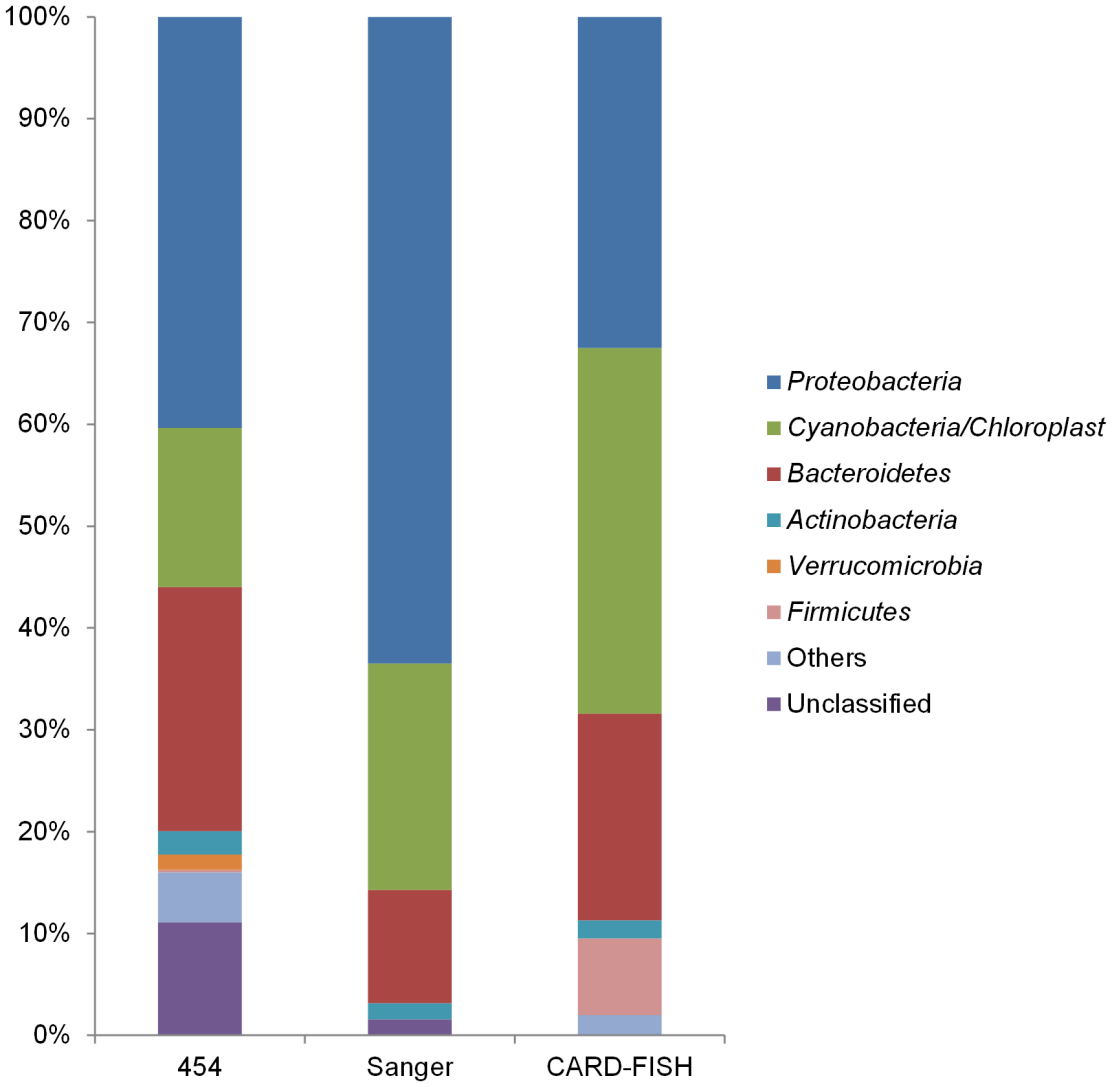
Microbial community composition at phylum level. Only phyla with abundance above 1% are shown.

Around 81.9±7.6% of DAPI-stained cells could be visualized by CARD-FISH with HRP-labeled oligonucleotide probe EUB338. By the applied protocol and stringency conditions, only very few cells appeared as positively hybridized by the probe ARCH915. The set of probes used to detect the major divisions within the *Bacteria* domain associated 98% of the EUB338 hybridized cells to known bacterial groups (Fig. 3). The negative control probe detected 0 to 2% of the DAPI-stained cells.

Among the *Proteobacteria* (32.5±10.5% of DAPI- stained cells), *Alpha-*, *Beta-* and *Gammaproteobacteria* accounted for 11.8±3.6, 5.8±2.0, and 12.8±2.8%, respectively. Only 1.2±0.9 and 0.9±1% of EUB338 stained cells were detected by probes for the *Delta*- and *Epsilonproteobacteria*. The LGC, HGC and CF319 stained cells (*Firmicutes*, *Actinobacteria* and *Cytophaga-Flavobacterium-Bacteroidetes* group, respectively) accounted for 7.5±2.9, 1.8±0.9 and 20.3±4.2%, respectively. Autotrophic pico-cyanobacteria were particularly abundant and represented 35.9±10.0% of DAPI-stained cells (Fig. 3).

### 454 16S amplicon pyrosequencing

Extracted DNA from snow sample amounted to 8.9 ng µl^−1^. From the initial data set containing 16914 reads (mean read length 453 bp), 16674 reads (mean read length 419 bp) were retained after filtering based on the quality of the barcode and primer sequence (barcode and primer sequence were removed in this step), 14657 reads (mean read length 404 bp) after basic and quality trimming, 14142 reads (mean read length 408 bp) after screening based on the alignment of the reads to the SILVA database. After denoising, those 14142 reads can be represented by 3772 unique reads. Applying UPARSE (combining chimera detection and OTU clustering) resulted in a total number of 820 OTUs representing 14043 reads with an average length of 420 bp (Table S1 in File S1). Next, the RDP classifier assigned those 820 OTUs to 303 different phylotypes (Table S2 in File S1). The obtained data covered a broad spectrum of known bacterial phyla, with the predominance of *Proteobacteria* (40.4%; 140 phylotypes) and *Bacteroidetes* (23.9%; 47 phylotypes), followed by *Cyanobacteria* (15.6%; 14 phylotypes), *Actinobacteria* (2.3%; 14 phylotypes) and *Verrucomicrobia* (1.5%; 15 phylotypes) (Fig. 3). The *Fusobacteria*, *Planctomycetes*, *Lentisphaerae*, *Firmicutes*, *Chlorobi*, *Tenericutes*, *Acidobacteria*, *Spirochaetes* and *Chloroflexi* were less abundant and were designated as “others” in Fig. 3. Most of the sequences that remained unclassified at the phylum level (11.1%) belonged to Phylotype_P001 (Table S3 in File S1).

**Figure 4:**
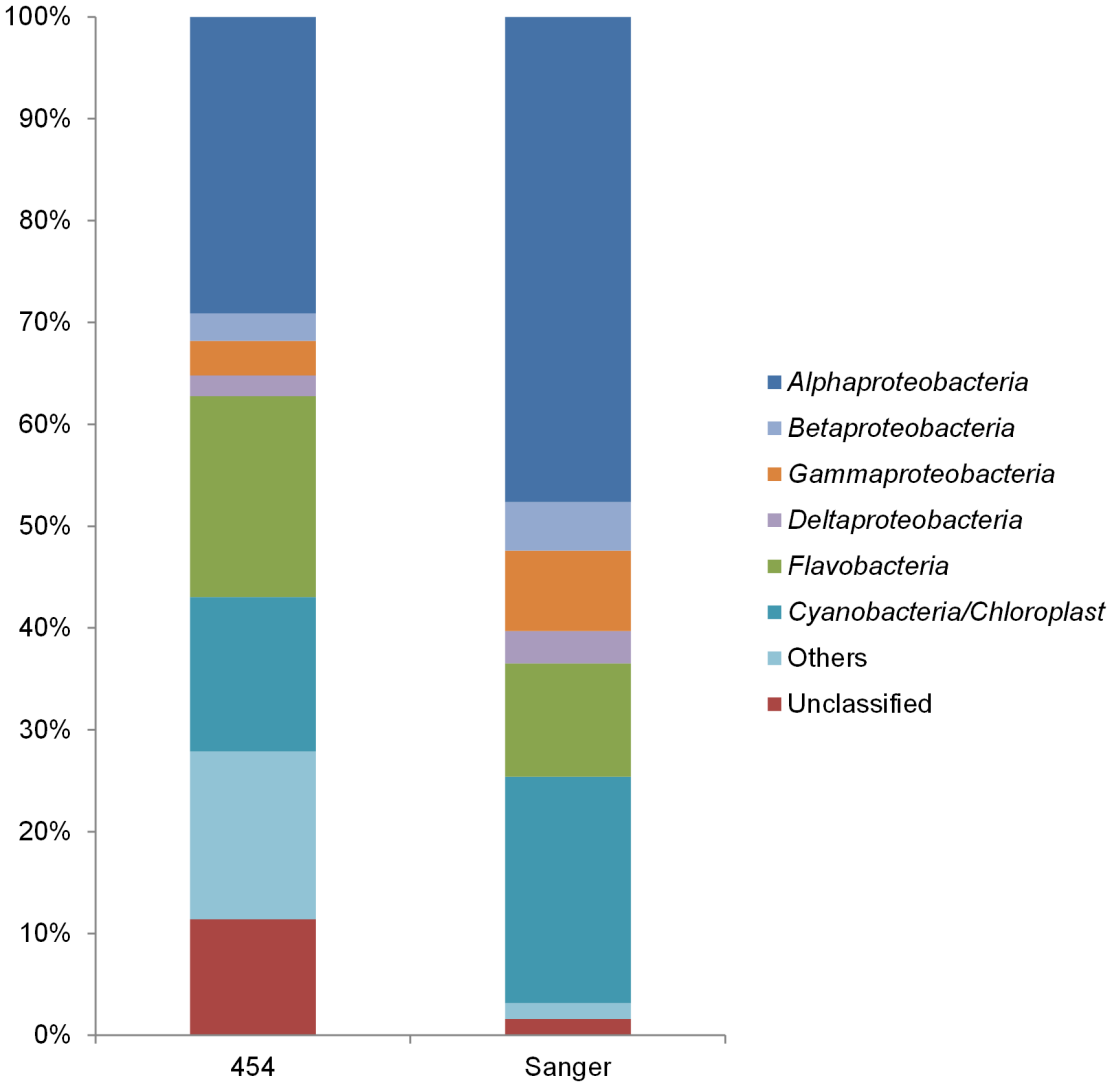
Microbial community composition at class level. Only classes with abundance above 1% are shown.

Among the *Proteobacteria*, members of the *Alpha-* (30.0%; 52 phylotypes), *Beta-* (2.8%; 15 phylotypes), *Gamma-* (3.5%; 40 phylotypes), *Delta-* (2.1%; 24 phylotypes), and *Epsilonproteobacteria* (1.9%; 7 phylotypes) were detected (Fig. 4, Tables S3 and S4 in File S1). The classified *Alphaproteobacteria* were mainly represented by *Kiloniellaceae* (7.5%; 3 phylotypes), *Rhodobacteraceae* (5.7%; 10 phylotypes) and *Rhodospirillaceae* (1.7%; 6 phylotypes). Almost half of the *Alphaproteobacteria* (40.7%) remained unclassified and most belonged to Phylotype_P002 (Table S3 in File S1). Also most of the *Gammaproteobacteria* remained unclassified, although 22.3% were SUP05-related (sulfur oxidizers). The classified *Betaproteobacteria* were mostly assigned to *Methylophilales* (*Methylophilaceae*), *Burkholderiales* (*Comamonadaceae*) and *Rhodocyclales* (*Rhodocyclaceae*) (Tables S3 and S4 in File S1). The order Sva085, *Desulfobacterales* (*Desulfobacteraceae* and *Desulfobulbaceae* families) were most abundant among *Deltaproteobacteria*. These families include sulfate-reducing organisms such as those belonging to the *Desulfotalea* genus (Phylotype_P088). Most *Epsilonproteobacteria* (96.6%) were affiliated to the *Campylobacterales* with the predominance of members of the genera *Arcobacter* and *Sulfurimonas* (Tables S3 and S4 in File S1).

Classified *Bacteroidetes* were predominantly affiliated to the *Flavobacteriaceae*. Approximately 70% of the *Cyanobacteria/Chloroplast* sequences were related to algal chloroplasts, including genera from *Chlorophyta*, *Cryptophyta*, *Stramenopiles*, *Haptophyceae* and *Streptophyta*. In addition, 27.2% of the *Cyanobacteria/Chloroplast* sequences were related to *Synechococcus*. Among *Actinobacteria*, *Acidimicrobiales* (OCS155 and C111) and *Microbacteriaceae* were most abundant. Among *Verrucomicrobia*, *Opitutae* (unclassified and *Puniceicoccaceae*) and *Verrucomicrobiaceae* were most abundant (Tables S3 and S4 in File S1).

### 16S rRNA gene clone library

Amplification of bacterial 16S rRNA genes was positive. However, no amplification of archaeal 16S rRNA genes was detected, thus supporting the outcome of the CARD-FISH analysis. The construction and screening of a 16S rRNA gene clone library (95 clones) yielded an initial data set containing 72 reads (mean read length 1078 bp). From this set, 63 reads (mean read length 924 bp) were retained after basic and quality trimming, and screening based on the alignment of the reads to the SILVA database (Table S1 in File S1). After denoising, those 63 reads can be represented by 59 unique reads. Applying chimera detection and OTU clustering resulted in a total number of 50 OTUs representing 63 reads with an average length of 923 bp (Table S1 in File S1). Next, the RDP classifier assigned those 50 OTUs to 25 different phylotypes (Tables S5 and S7 in File S1). The obtained data covered four bacterial phyla, with the predominance of *Proteobacteria* (63.5%; 15 phylotypes) and *Cyanobacteria/Chloroplast* (22.2%; 4 phylotypes), followed by *Bacteroidetes* (11.1%; 4 phylotypes) and *Actinobacteria* (1.6%; 1 phylotype) (Fig. 3). The classified *Alphaproteobacteria* were mainly represented by *Pelagibacteraceae* (25.4%), and *Rhodobacteraceae* (6.4%). The classified *Gammaproteobacteria* included *Aeromonas*. The *Bacteroidetes* were affiliated to the *Cryomorphaceae* and *Flavobacteriaceae* families. Fifty per cent of the *Cyanobacteria/chloroplast* sequences were related to algal chloroplasts (Table S7 in File S1).

### Comparison of different approaches

**Figure 5:**
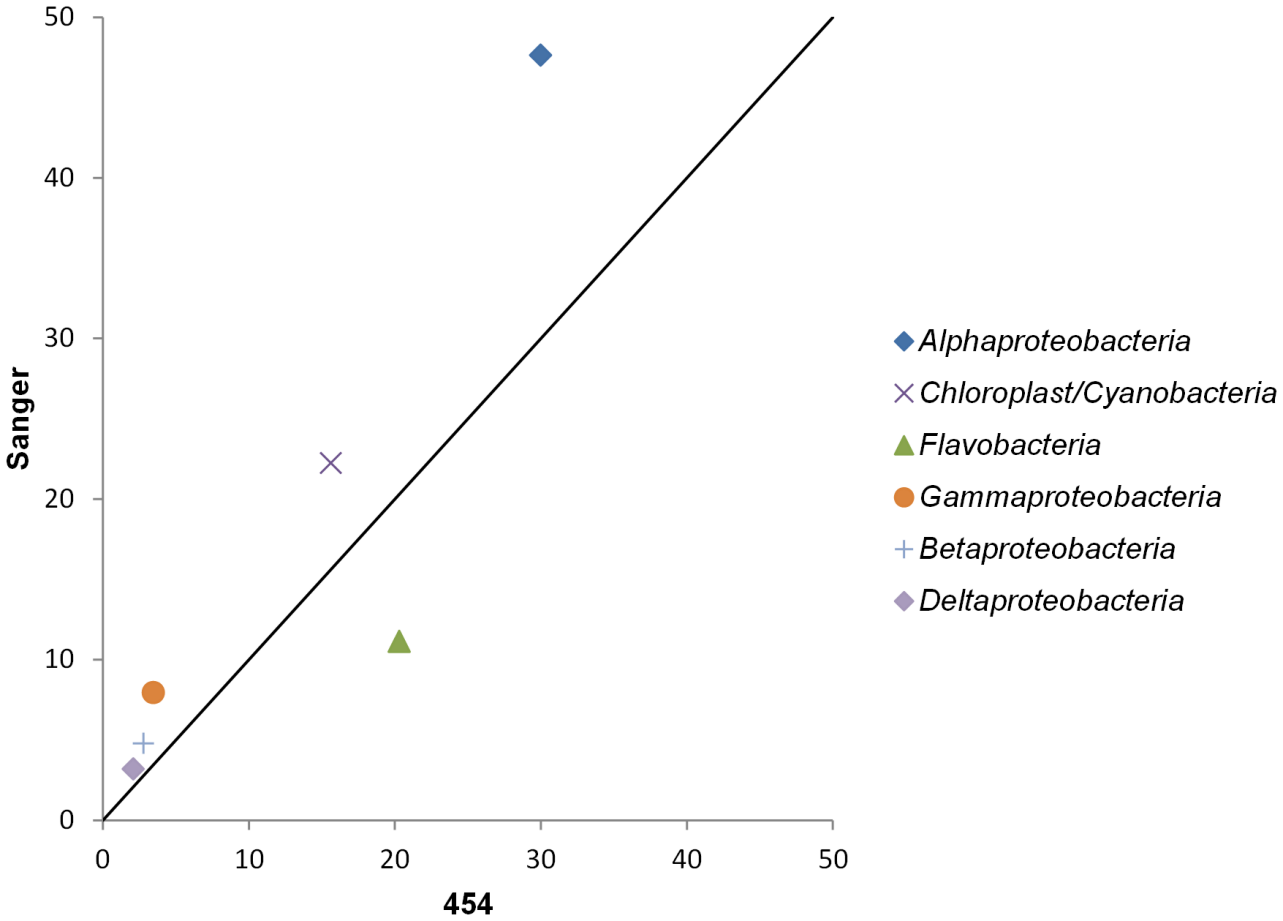
Relative contribution of specific bacterial classes to the bacterial community. 454 16S amplicon pyrosequencing *versus* 16S rRNA gene clone library.

Overall a reasonable agreement between the composition of bacterial phyla were found with the three techniques (Fig. 3). Also at class level, a good agreement was found with both sequencing techniques (Figs. 4 and 5). However, some differences were found between the contribution of specific bacterial groups to the total community for instance for *Flavobacteria* and *Cyanobacteria*. Nevertheless, only the increased contribution of *Cyanobacteria* in the clone library was found to be statistically deviating from the results based on the pyrosequencing reads (p-value <0.05 using the hypergeometric distribution). As the amount of extracted DNA was limited, our outcome could be biased by contaminating bacterial DNA in reagents [40], [41]. However, the comparable outcome of both sequencing techniques, which used different types of polymerase and reagents, indicated that the overall community composition is unlikely to be attributed to contamination.

## Discussion

This study represents the first report on the microbiome of the surface snow surrounding the Research Base “Concordia” on the high Antarctic Plateau (3233 m above the sea level at Dome C on the Eastern Antarctic plateau). This unique environment is used (next to glaciology, atmospheric sciences, astronomy, etc.) as a test bed for long-duration spaceflights to study the physiologic and psychological adaptation to isolated environments [14]. In addition, Antarctica is seen as an analogue of extraterrestrial environments [3].

Bacterial densities estimated by direct epifluorescence microscopy and flow cytometry are in line with previous reports for South Pole snow (200 to 5000 cells ml^−1^) [7], but were two orders of magnitude lower than in snow at Ny-Alesund (Spitzberg) (6×10^4^ cells ml^−1^) [10], on the Tibetan Plateau (0.68 to 720×10^3^ cells ml^−1^) [5], or in Alpine sites (1.1×10^4^ cells ml^−1^) [42]. Moreover, about 30% of the total counted prokaryotes showed the typical autofluorescence signature of phycocyanin- and phycoerythrin-containing *Cyanobacteria*, as estimated by either flow cytometry (26.5%) or epifluorescence microscopy (35.9%). This finding was supported by the identification of *Cyanobacteria* by both sequencing techniques.

Overall, the bacterial community composition in surface snow surrounding the Concordia Base at Dome C was similar to that reported in glaciers, snow, lake ice, sea ice, and atmospheric clouds [4]. Although the site is remote from the sea, a strong influence from the marine environment seems to exist with *Proteobacteria* being the dominant members, followed by *Bacteroidetes* and *Cyanobacteria* [43]. Allochthonous input of marine microorganisms and other particulate organic matter are introduced by atmospheric circulation and deposition [7], [13], [44], [45]. Free-living microorganisms have high dispersal rates and can be transported over very long distances because of their microscopic size and resistance to environmental stressors [4], [46]. However, we cannot *a priori* exclude the possibility that a minor part of microorganisms could come from human activity even when the chosen sampling site was two kilometers from Concordia Station in the restricted access “Clean Area” zone.

**Figure 6:**
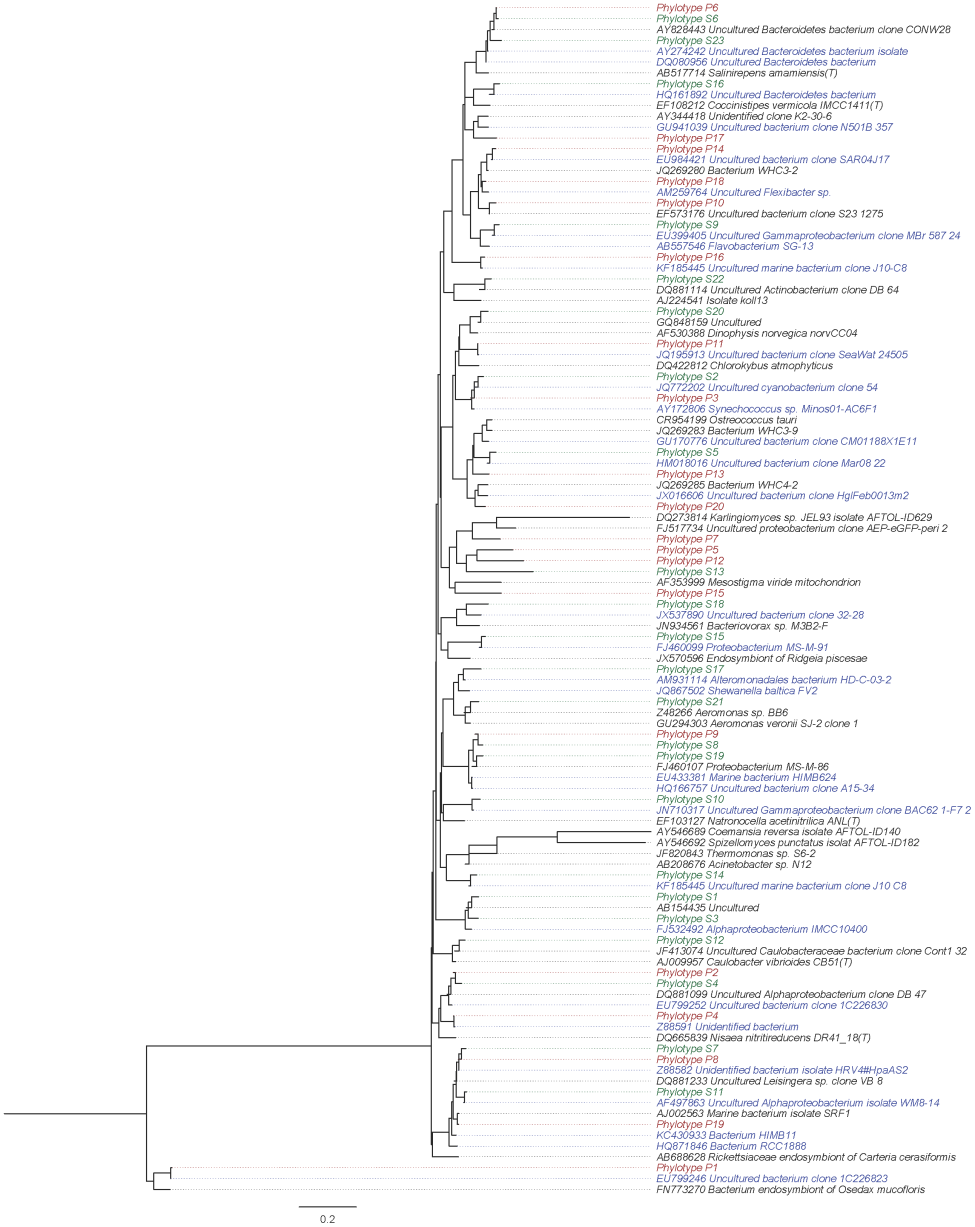
Phylogenetic relationship of snow phylotypes and their closest relatives. The closest relatives to the phylotypes from the 16S rRNA gene clone library (green; Phylotype S) and 454 16S amplicon pyrosequencing (15 most abundant) (red; Phylotype P) were retrieved from the RDP cultured and the greengenes dataset and compiled to calculate an unrooted phylogenetic tree via mothur. All relatives isolated from marine origin are shown in blue.

Among the *Alphaproteobacteria*, *Kiloniellaceae* and *Rhodobacteraceae* were the most abundant groups. Many were phylogenetically related to sequences retrieved from marine origin (Fig. 6). Such a family exhibits a variety of growth modes like photo- and chemo-heterotrophic growth under light anoxic conditions and dark micro-oxic to oxic conditions, respectively, as well as photolithoautotrophic growth. Different members of the *Rhodobacteraceae* (*Amaricoccus, Anaerospora, Loktanella, Octadecabacter, Paracoccus, Phaeobacter and Thalassobius*) were detected with 454 16S amplicon pyrosequencing. Nevertheless, almost all of the *Kiloniellaceae* and 75.5% of the *Rhodobacteraceae* reads remained unaffiliated.

The *Gamma-*, *Beta-*, *Epsilon*- and *Deltaproteobacteria* were detected at lower levels (454 16S amplicon pyrosequencing). Among the *Epsilonproteobacteria*, the most represented genera were *Arcobacter* and *Sulfurimonas*. *Arcobacter* is a unique genus harboring species that can grow microaerobically, occupying various ecological niches, e.g. in association with animals (and humans) and other species that are free-living and found in seawater [47]. Among the classified *Gamma- and Deltaproteobacteria*, members related to the sulfur and nitrogen cycles were retrieved.

Members of the *Bacteroidetes*, predominantly *Flavobacteriaceae*, represented a significant fraction of the bacterial community in the surface snow at Dome C. Members of *Flavobacteriaceae*, originate from marine environments and are important for the mineralization of organic matter in marine ecosystems [48] (Fig. 6).

Another main group identified was the *Cyanobacteria* class (dominated by the *Synechococcus* genus). Marine synechococci are ubiquitously distributed throughout the oceans and can be found in any ecosystem type up to the polar circle [49] including extreme regions like the Mount Everest (in the snow at 6 500 m altitude) [50] and in extensive snow cover on the Byers Peninsula (Livingston Island, maritime Antarctica) [51]. The *Cyanobacteria* are organisms that occur in most sun-exposed ecosystems on Earth and are frequently found in ice as well as cold desert habitats [52]. They are relatively rare in non-stable cryo-ecosystems as melting snow, but have been recorded in glacier snow from several areas of the world [45], [53].

The phylogenetic affiliation also revealed the presence of eukaryotic (chloroplasts) taxa. The closest relatives of many phylotypes were related to marine environments. In particular, *Chlorophyta* and *Cryptophyta* were identified in both the cloning and the pyrosequencing dataset. This data is supported by the cytometric and microscopic observation of picoeukaryotes (Fig. 2).

The more than 300 phylotypes identified in this work represent a significant diversity taking into account the environmental conditions and remoteness of the site. The dataset covers the majority (98.2% coverage) of the diversity and is in line with previous reports, which include heterotrophic bacteria, cyanobacteria and eukaryotes, with many of them that are related to known psychrophilic and psychrotolerant species [54].

**Figure 7:**
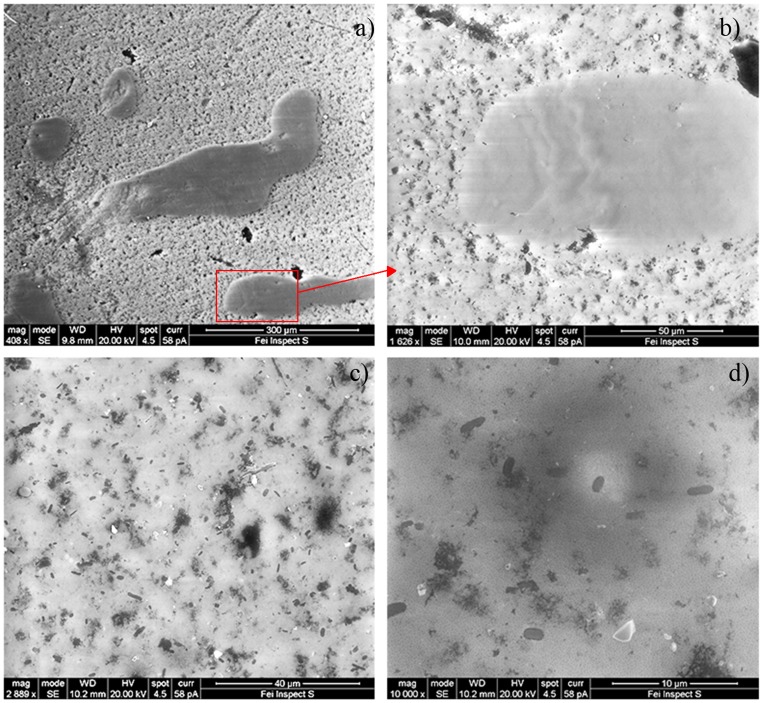
SEM pictures of bacteria from Dome C surface snow. Pictures are at increasing magnification from a) to d).

Although our results provide no direct evidence for possible activity of the depicted bacterial community, some may be indicative for activity. The community could be organized in a patchy biofilm structure suggested by the presence of exopolysaccharide-like debris on the DAPI-stained filters (not shown) and by scanning electron microscopy (Fig. 7). Furthermore, exopolysaccharides are thought to be a primary protection mechanism by reducing water loss and by restricting ice crystal formation to sites outside the cells [53]. In addition, *Cyanobacteria*, detected as auto-fluorescent cells via flow cytometry, as well as bacteria involved in carbon, nitrogen and sulfur cycles, were identified.

It has been reported that microbial abundance in the snowpack is usually positively correlated with Ca^2+^ concentrations, serving as a proxy for dust [54]. Moreover, microorganisms in dry polar snow were shown to be involved in active exchange of reactive nitrogen species with the atmosphere and were thus contributing to biogeochemical cycling at low temperature [55]. Despite the low temperatures and limited liquid water supply, microbes inhabiting the snow cover seemed to constitute a dynamic community, as it was recently suggested by the observation of a link between microbial community structure and snow chemistry [56]. Survival of prokaryotes and unicellular eukaryotes has been demonstrated down to −18°C in unfrozen hypersaline polar lakes, as well as metabolism of bacteria from snow at −17°C and from permafrost at −20°C [7], [57]. However, the lower temperature limit for cell growth and division remains unclear. At least for nitrification, some reports exist on activities at temperatures close to the Concordia surface snow at sampling time. Miteva and colleagues [58] observed experimentally nitrification by *Nitrosomonas cryotolerans* in solutions frozen at −32°C. Price and Sowers [59] reported evidence for metabolically active bacteria and fungi at temperatures down to −40°C. Finally, Sowers [60], who studied the N_2_O in Antarctic ice cores, suggested that the recorded artifacts correlated with high concentrations of dust and high bacterial counts could be explained by the presence of metabolically active bacteria, presumably brought to the ice caps with the dust, that had produced sufficient N_2_O over the years to alter the composition of the archived air.

## Conclusions

This study represents the first investigation focused on the microbiome of the surface snow surrounding the Research Base “Concordia” on the high Antarctic Plateau. In such remote and extreme environment, a strong influence from the coastal areas seems to exist with marine *Proteobacteria* (i.e. *Kiloniellaceae* and *Rhodobacteraceae*) representing the dominant members within the microbial community, followed by *Bacteroidetes* and *Cyanobacteria*. Thus, our findings support the evidence that polar microorganisms should be considered not only as deposited airborne particles, but also as an active component of the snowpack ecology.

## Supporting Information

File S1 **Output from the sequencing data processing: descriptive statistics, classification and number of phylotypes in the 16S rRNA gene clone library and 454 16S amplicon pyrosequencing data sets.**
(XLSX)
